# Haematology in Africa

**DOI:** 10.1111/j.1365-2141.2011.08763.x

**Published:** 2011-07-05

**Authors:** Lucio Luzzatto, Foluke Fasola, Léon Tshilolo

**Affiliations:** 1 Istituto Toscano TumoriFirenze, Italy; 2 Department of Haematology, College of Medicine, University of IbadanIbadan, Nigeria; 3 Centre Hospitalier Monkole/Centre de Formation et d'Appui Sanitaire (CEFA)Kinshasa, DR Congo

**Keywords:** haematology, Africa, developing world

All diseases result from our genes and from the environment. This is conventional teaching for first year medical students: and if the medical school is in Africa it is hard to find a better example than blood diseases, given that in Africa, on one hand, haemoglobinopathies affect millions of people (WHO Working Group, 1982), while on the other hand, malaria, hookworm and nutritional deficiency (folate, or iron or both) are the main causes of anaemia (Boele van Hensbroek *et al*, 2010). In medical school we must also deal with the role of Darwinian selection in evolution. This is a central thread throughout biology, and almost everyone agrees it applies extensively also to the human species: yet, when it comes to giving an example, whether the class is held in Africa or elsewhere, the teacher will fall back on how the haemoglobin S gene has been selected in tropical areas through the relative resistance it confers to heterozygotes against *Plasmodium falciparum* (see Fig 1 and Luzzatto, 1979).

**Fig. 1:**
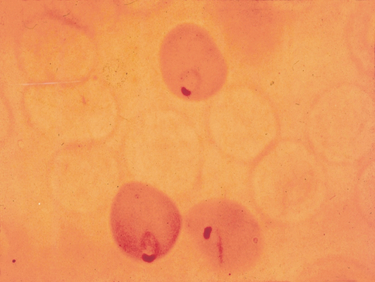
*Plasmodium falciparum* infection in a girl heterozygous for Glucose-6-phosphate dehydrogenase (G6PD) deficiency. The blood film was stained by a procedure that causes lysis of G6PD-deficient red cells, producing their ghost-like appearance. As G6PD deficiency heterozygosity is a somatic cell mosaic as a result of X chromosome inactivation it was possible to show by this procedure that *P. falciparum* parasitized red cells are predominantly G6PD normal (see Luzzatto *et al*, 1969).

This paper is not a review, but a commentary; given that Africa is a vast and variegated continent, we have concentrated on its tropical area – or, as from a phrase customary during the last century, Africa ‘south of the Sahara and north of the Zambesi’. So what are the issues faced by the practicing haematologist in Africa today? The short answer is, exactly the same as those faced by a haematologist in any other part of the world: only a little more complicated, for two reasons. (i) Tropical diseases are not in lieu, but in addition to those that prevail in temperate climates. It has long been the practice anywhere within the rain forest belt that, when a newly admitted patient has a high fever the question is not whether he needs treatment for malaria, but whether the fever is due ‘just’ to malaria (Esan, 1975) or to other additional factors. Example of co-morbidity we remember include a patient who had HbSC disease and chronic myeloid leukaemia (see also (Rosner & Grünwald, 1989); patients who had both tuberculosis and lymphoma (see Omoti *et al*, 2009); a patient with sickle cell anaemia who was at risk of dying from malaria (see McAuley *et al*, 2010); a young woman with anaemia in pregnancy who was found to have paroxysmal haemoglobinuria and developed fulminating amoebiasis (Oni *et al*, 1970). In the Northern part of the world co-morbidity is quoted often as a troublesome component common in oncological practice in the elderly; in Africa co-morbidity can be the rule rather than the exception at any age. (ii) Many patients need haematological attention for problems that may have a precise aetiology, but that have in fact developed essentially as a result of socio-economic reasons, as has also happened abundantly elsewhere. For instance, hookworm anaemia is a specific diagnosis: but when Italian workers digging the Gotthard tunnel in the late 19th century became paler and paler until they died it was called *miners’ anaemia* or *tunnel disease.* Once Camillo Bozzolo, Edoardo Perroncito, and Luigi Pagliani correctly surmised that the spread of the disease had something to do with the workers wearing worn-out shoes and being forced to defecate inside the 15 km tunnel, the problem was quickly solved (Parona, 1894). Today in Africa the same condition, hookworm anaemia, could be called the *barefoot farmer’s anaemia*: should it be treated by the administration of mebendazole and iron or would it be better for farmers to have proper footwear? Today in Africa the spectrum of blood diseases is largely similar to that of the rest of the world, but disease presentation may be considerably modified by socio-economic and cultural factors (see Figs 2 and 3). In practice, this often means late presentation with advanced disease, and in some cases even mutilation, particularly with neoplastic disease. The same factors also influence the rate of patients who discharge themselves or their children against medical advice, and the degree of compliance of patients with treatment protocols and follow up (Diagne *et al*, 2003): in these respects the role of social workers is paramount and their services ought to be much enhanced.

**Fig. 2:**
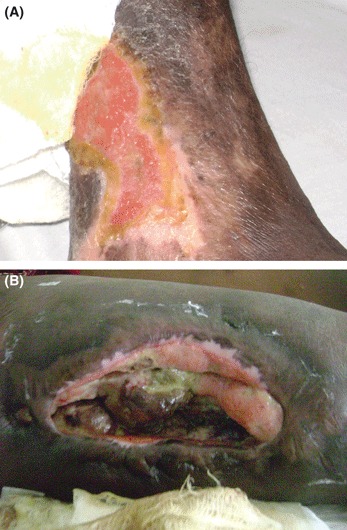
Chronic complications of sickle cell anaemia. (A) Typical ulcer of 5 years duration on the lateral aspect of the right ankle in a 19-year-old male. Upon regular conventional conservative care the ulcer appeared to have healed on two occasions, but unfortunately it broke down again after minor trauma. A skin autograft was then carried out but failed. (B) Atypical ulcer of 3 years duration in the anterior aspect of the right thigh of a 26-year-old woman. The ulcer developed following repeated intramuscular injections of pentazocine, administered as a pain-killer by a nurse and by the patient herself. When self-care of the wound by dressing became unmanageable the patient saw a specialist and subsequently travelled to India twice for skin grafting, but the procedure failed on both occasions. It is seen that the ulcer in panel B is much deeper than that shown in panel A, reflecting a different pathogenesis. Whereas the ulcer in panel A was a direct complication of sickle cell anaemia, the ulcer in panel B can be regarded as an indirect self-inflicted consequence: painful attacks prompted administration of analgesics, and the patient, who was at the time a University student, opted for self-medication in order to avoid frequent visits to the hospital, thus saving time and money. The patient is still suffering from the consequences of ill-advised inappropriate management, but this ultimately still goes back to her underlying sickle cell anaemia. A significant impact of socio-economic status on the clinical course of sickle cell anaemia has been documented in Nigeria (Okanyi & Akinyanju, 1993).

**Fig. 3:**
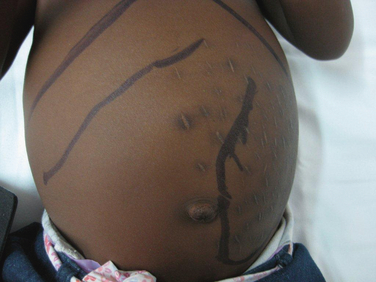
Splenomegaly in Africa: different causes and different treatments. This 9-year-old girl had a massive spleen. A traditional doctor had applied tattoos or scarifications that did not control the condition. A likely diagnosis is hyper-reactive malarial splenomegaly (Bedu-Addo & Bates, 2002), and it is hoped that the condition will respond to prolonged administration of antimalarial drugs (Sagoe, 1970).

Co-morbidity problems and other complex clinical situations can be solved by clinical acumen sharpened by experience, diligent review of the literature, appropriate interactions with colleagues, and obstinate perseverance. On the other hand, dealing with major public health problems that manifest through a high prevalence of various types of anaemia (see Table I) is a tall order: what then, in this respect, is the role of a haematologist? It would be certainly wrong to say that a haematologist should not be concerned with the matter; but it would be equally wrong to blame haematologists for the death of children with malaria and severe anaemia or of women with acute blood loss from *placenta praevia* who have not been transfused promptly enough. We do have an obligation to pinpoint these problems, but this has been done in the literature of the past several decades: it will not help if we continue to designate problems as *medical* when they are, in fact, economic. We must recognize that such problems ought to be resolved by adopting appropriate public health policies, in particular by improvement of the infrastructure; and that ultimately the basis for such decisions is the will or at least the willingness to make political choices.

**Table I:** Common anaemias at the border between haematology and public health

Designation	Aetiopathogenesis (main factor)	‘Real’ cause	Standard therapy	Long-term management
Hookworm anaemia	Chronic blood loss from infestation with *Anchilostoma duodenale*	Poor hygiene/poverty	Mebendazole, oral iron	Preventing worm infestation
Malarial anaemia	Recurrent infection with *Plasmodium falciparum* and other *Plasmodia*	High rate transmission of malaria by *Anophelines*	Anti-malarial chemotherapy	Eradication of malaria transmission
Severe anaemia in pregnancy	Increased folate requirement	Poor folate intake	Folic acid, folinic acid	Improving nutrition
Severe post-partum anaemia	Ante-partum or post-partum haemorrhage	Inadequate ante-natal or obstetric care	Blood transfusion	Improving ante-natal care

Nowadays, much of what a haematologist is called upon to do has the onco- prefix, and Africa is no exception. It is pertinent to recall that our understanding of lymphoma owes much to an African disease which, following the painstaking epidemiological studies conducted half a century ago by Dennis Burkitt (Burkitt, 1962), carries his name. It was on biopsy tissue from East Africa and from cell lines grown in Ibadan (Osunkoya & Mottram, 1967) that the t(8;14) translocation was discovered in Burkitt lymphoma. The subsequent identification of *IGH* (previously *IgH*) and *MYC* (previously *c-myc*) as partner genes that become juxtaposed and dysregulated as a result of this translocation spelled a new paradigm in the mechanism of oncogenesis (Klein, 1983). Today, accurate diagnosis of most types of lymphoma and of acute leukaemia can be done in most haematology departments in Africa; however, with a few exceptions, this is followed all too often by the frustrating feeling that one cannot provide optimal treatment: sometimes because of limited access to the necessary drugs, sometimes because of inadequate facilities (e.g. for bone marrow transplantation), sometimes both. However, complaining about shortages will not get us anywhere; rather, it would be probably ideal if each department worked out, for internal consumption, a list in three sections (see Table II): (A) Conditions we can deal with adequately. (B) Conditions for which we offer treatment that may not be optimal in 2011, but is nevertheless of proven efficacy. In this respect, treatment approaches using less intense and less costly multi-agent chemotherapy are sometimes successfully introduced with encouraging results (Fasola *et al*, 2002; Hesseling *et al*, 2008) (C) Conditions for which we cannot offer adequate treatment; these cases, i.e., those who can afford it and especially potentially curable patients, must be recommended to attend elsewhere for treatment. Clearly the contents of the three sections will vary from place to place: but it would be an advantage for both doctors and patients to have taken the deliberate step of setting and stating in advance a policy to which one can refer, even though it is hoped that adjustments will be needed frequently.

**Table II:** Management of relatively common conditions in onco-haematology: a few examples

Category	Condition	Basic requirements for treatment	Comments
A	Chronic lymphocytic leukaemia	Fludarabine, rituximab, chlorambucil	Typing for risk stratification may be out-sourced
	Polycythaemia rubra vera	Phlebotomies; hydroxycarbamide	
	Burkitt lymphoma	Cyclophosphamide, vincristine, doxorubicine, prednisone	
B	Hodgkin lymphoma	ABVD, BEACOPP; radiotherapy	Salvage therapy may not be available
	Myeloma	Melphalan, prednisone, auto-BMT	Second and third line drugs may not be available
	Common acute lymphoblastic leukaemia	Vincristine, prednisone, intrathecal MTX	
C	Chronic myeloid leukaemia	Imatinib; allogeneic BMT	Imatinib is expensive; allogeneic BMT may not be available
	Acute leukaemia in adults	Several drugs, allogeneic BMT	

ABVD, doxorubicin, bleomycin, vinblastine, dacarbazine; BEACOPP, bleomycin, etoposide, adriamycin, cyclophosphamide, vincristine, procarbazine, prednisone; MTX, methotrexate; BMT, bone marrow transplantation.

It must be acknowledged that, in many countries, haematology has become sub-specialized to the point of fragmentation, with a haematologist who mainly performs transplants being hardly likely to ever see a patient with haemophilia, and with the myeloma expert having nothing to do with blood transfusion. In Africa the general haematologist species is not yet extinct, and contributions in all of these areas have come from general haematologists (e.g. see Essien *et al*, 1970; Worlledge *et al*, 1974; Kasili, 1976; Williams, 1985): this means greater demands on one’s time in order to keep up to date, but also a command of a wider field, with a combination of clinical and laboratory skills that can be turned to one’s advantage. Many haematologists in many parts of the world have also given up looking down the microscope, but not so in Africa where more limited resources must be exploited fully: and fortunately a good microscope does not obsolesce as quickly as a flow cytometry machine. So let us not underestimate how many blood conditions can be diagnosed or at least suspected just by looking carefully at a blood film; it is possible that, in the future, selected African haematologists will be needed to teach blood morphology in other continents!

Against this background, if in a brief commentary we are to single out one disease as the *Leitmotiv* of African haematology, it must be undoubtedly the haemoglobinopathies, and specifically sickle cell disease. Last year was the centennial anniversary of the description of sickle cells by Herrick (1910). During these one hundred years there have been tremendous advances in understanding the disease, but for the first three quarters of that century there has been remarkably little progress in improving its management. Then, three developments occurred: (i) prenatal diagnosis (Old *et al*, 2000), (ii) bone marrow transplantation (Vermylen *et al*, 1994; Walters *et al*, 1996) and (iii) hydroxycarbamide (HC, previously termed hydroxyurea) (Charache*, et al* 1995; Zimmerman *et al*, 2004). For each one of these developments there are *pros* and *cons*, including difficult clinical decisions and weighty ethical issues, which are not expanded upon in this article. But, we must ask a simple question: to what extent have these advances been of benefit to patients in Africa, where the large majority (estimated 8 million) of patients with sickle cell disease live? The facilities required for bone marrow transplantation are considerable, but they are certainly present in South Africa (Wood *et al*, 2009); the facilities required for prenatal diagnosis are relatively modest, especially since this has shifted from haemoglobin-based to DNA-based (Adewole *et al*, 1999). As for HC, the daily cost of this drug per patient in the UK is about 20 p: unfortunately this cost is higher in Africa, where it is estimated that, in many countries, no more than 1% of patients with sickle cell disease receive HC; and this to us is a shame. Yet, significant background information is available: a search in PubMed for *sickle cell disease AND Africa* yields no less than 1386 hits, about one-tenth of the total *sickle cell disease* hits (including, for instance, Konotey-Ahulu, 1974; Bienzle *et al*, 1975; Akinyanju *et al*, 2005; Labie & Elion, 2010; Makani *et al*, 2011). It is true that, although HC is a reversible inhibitor of deoxyribonucleoside diphosphate reductase and *not* an alkylating agent, questions are still being asked about the possible risk of cancer from this drug: however, there is no theoretical or empirical reason why HC should be less beneficial or less safe for African patients compared to other patients elsewhere (Jones *et al*, 2010; Svarch *et al*, 2006; Steinberg *et al*, 2010).

What is the way forward? Given that little research support is available, should we concentrate more on laboratory experiments, clinical data collection, outcome research or educating the public? As stated at the beginning of this article, we must strike a balance between tackling problems in which haematologists can make a difference and taking on others that are much larger than all haematology departments in the world pooled together. To treat an individual with folate deficiency or with hookworm anaemia is easy and gratifying; but unless poverty is relieved it will be a drop in the ocean, and to relieve poverty we must – regardless of being haematologists – mitigate as citizens or go into politics. When poverty is relieved these diseases will disappear, just like protein-calorie malnutrition in children. The practice of haematology in an African country, like in any other country, is part and parcel of a national structure that depends on a political system: inevitably this has a strong impact on the delivery of optimal haemato-oncology treatments to patients. In Africa, this challenge is initially tied up with the availability of, and access to, the necessary drugs; and it is compounded by the emergence of human immunodeficiency virus (HIV) as a major threat to blood safety (Lefrère *et al*, 2011).

In the meantime, we have an obligation to fulfill our specific professional commitments. There is no doubt that international initiatives could help, as indicated by a recent report (Odame, 2010); at the same time, international partnerships could also provide the impetus for research and help to target resources towards the goal of improving patient care. Let us start with a campaign to provide every African who has sickle cell disease with folic acid, antimalarial prophylaxis and, whenever indicated, HC.

## References

[b1] Adewole T, Olukosi YA, Disu F, Akinde JA, Adesemoye E, Akinyanju OO, Afonja OA (1999). Application of polymerase chain reaction to the prenatal diagnosis of sickle cell anaemia in Nigeria. West African Journal of Medicine.

[b2] Akinyanju OO, Otaigbe AI, Ibidapo MOO (2005). Outcome of holistic care in Nigerian patients with sickle cell anaemia. Clinical and Laboratory Haematology.

[b3] Bedu-Addo G, Bates I (2002). Causes of massive tropical splenomegaly in Ghana. Lancet.

[b4] Bienzle U, Sodeinde O, Effiong CE, Luzzatto L (1975). G6PD deficiency and sickle cell anemia: frequency and features of the association in an African community. Blood.

[b5] Boele van Hensbroek M, Calis JC, Phiri KS, Vet R, Munthali F, Kraaijenhagen R, van den Berg H, Faragher B, Bates I, Molyneux ME (2010). Pathophysiological mechanisms of severe anaemia in Malawian children. PLoS ONE.

[b6] Burkitt D (1962). A “tumor safari” in East and Central Africa. British Journal of Cancer.

[b7] Charache S, Terrin ML, Moore RD, Dover GJ, Barton FB, Eckert SV, McMahon RP, Bonds DR, the Investigators of the Multicenter Study of Hydroxyurea in Sickle Cell Anemia (1995). Effect of hydroxyurea on the frequency of painful crises in sickle cell anemia. New England Journal of Medicine.

[b9] Diagne I, Diagne-Gueye ND, Signate-Sy H, Camara B, Lopez-Sall P, Diack-Mbaye A, Sarr M, Ba M, Sow HD, Kuakuvi N (2003). [Management of children with sickle cell disease in Africa: experience in a cohort of children at the Royal Albert Hospital in Dakar]. Medecine tropicale.

[b10] Esan GJF (1975). Haematological aspects of malaria. Clinics in Haematology.

[b11] Essien EM, Folami AO, Luzzatto L (1970). Haemophilia in Nigeria. Tropical and geographical medicine.

[b12] Fasola FA, Shokunbi WA, Falade AG (2002). Factors determining the outcome of management of patients with Burkitt’s lymphoma at the University College Hospital Ibadan, Nigeria – an eleven year review. The Nigerian postgraduate medical journal.

[b13] Herrick J (1910). Peculiar elongated and sickle-shaped red blood corpuscles in a case of severe anemia. Archives of internal Medicine.

[b14] Hesseling P, Molyneux E, Tchintseme F, Welbeck J, McCormick P, Pritchard-Jones K, Wagner H-P (2008). Treating Burkitt’s lymphoma in Malawi, Cameroon, and Ghana. Lancet Oncology.

[b8] Jones AP, Davies SC, Olujohungbe A (2010). Hydroxyurea for sickle cell disease. Cochrane database of systematic reviews (Online).

[b15] Kasili E (1976). Current trends in chemotherapy of leukaemias lymphomas and multiple myeloma. East African Medical Journal.

[b16] Klein G (1983). Specific chromosomal translocations and the genesis of B-cell-derived tumors in mice and men. Cell.

[b17] Konotey-Ahulu FI (1974). The sickle cell diseases. Clinical manifestations including the “sickle crisis”. Archives of internal medicine.

[b18] Labie D, Elion J (2010). Le probléme de la drepanocytose en Afrique. Médecine tropicale (Marseille).

[b19] Lefrère J, Dahourou H, Dokekias AE, Kouao MD, Diarra A, Diop S, Tapko JB, Murphy EL, Laperche S, Pillonet J (2011). Estimate of the residual risk of transfusion-transmitted human immunodeficiency virus infection in sub-Saharan Africa: a multinational collaborative study. Transfusion.

[b20] Luzzatto L (1979). Genetics of red cells and susceptibility to malaria. Blood.

[b21] Luzzatto L, Usanga FA, Reddy S (1969). Glucose-6-phosphate dehydrogenase deficient red cells: resistance to infection by malarial parasites. Science.

[b22] Makani J, Cox SE, Soka D, Komba AN, Oruo J, Mwamtemi H, Magesa P, Rwezaula S, Meda E, Mgaya J, Lowe B, Muturi D, Roberts DJ, Williams TN, Pallangyo K, Kitundu J, Fegan G, Kirkham FJ, Marsh K, Newton CR (2011). Mortality in sickle cell anemia in Africa: a prospective cohort study in Tanzania. PLoS ONE.

[b23] McAuley CF, Webb C, Makani J, Macharia A, Uyoga S, Opi DH, Ndila C, Ngatia A, Scott JA, Marsh K, Williams TN (2010). High mortality from *Plasmodium falciparum* malaria in children living with sickle cell anemia on the coast of Kenya. Blood.

[b24] Odame I (2010). Developing a global agenda for sickle cell disease: report of an international symposium and workshop in Cotonou, Republic of Benin. American Journal of Preventive Medicine.

[b25] Okanyi C, Akinyanju OO (1993). The influence of socio-economic status on the severity of sickle cell disease. The African journal of medical sciences.

[b26] Old J, Petrou M, Varnavides L, Layton M, Modell B (2000). Accuracy of prenatal diagnosis for haemoglobin disorders in the UK: 25 years’ experience. Prenatal Diagnosis.

[b27] Omoti CE, Olu-Eddo AN, Nwannadi AI (2009). Co-existence of TB and adult haematological cancers in Benin City, Nigeria. Tropical doctor.

[b28] Oni SB, Osunkoya BO, Luzzatto L (1970). Paroxysmal nocturnal hemoglobinuria: evidence for monoclonal origin of abnormal red cells. Blood.

[b29] Osunkoya BO, Mottram FC (1967). Formal discussion: observations on the establishment of Burkitt’s tumor lymphoblasts in serially propagated cultures. Cancer research.

[b30] Parona C (1894). L’elmintologia italiana da’ suoi primi tempi all’anno 1890: storia, sistematica, corologia e bibliografia.

[b31] Rosner F, Grünwald HW (1989). Chronic granulocytic leukemia in a patient with hemoglobin SC disease. American Journal of Hematology.

[b32] Sagoe A (1970). Tropical splenomegaly syndrome: long-term proguanil therapy correlated with spleen size, serum IgM and lymphocytic transformation. British Medical Journal.

[b33] Steinberg MH, McCarthy WF, Castro O, Ballas SK, Armstrong FD, Smith W, Ataga K, Swerdlow P, Kutlar A, DeCastro L, Waclawiw MA (2010). The risks and benefits of long-term use of hydroxyurea in sickle cell anemia: a 17.5 year follow-up. American Journal of Hematology.

[b34] Svarch E, Machin S, Nieves RM, Marcia de Reyes AG, Navarrete M, Rodriguez H (2006). Hydroxyurea treatment in children with sickle cell anemia in Central America and the Caribbean countries. Pediatric Blood Cancer.

[b35] Vermylen C, Cornu G, Ferster A, Sariban E, Pinkel D, Garfunkel JM (1994). Bone marrow transplantation for sickle cell anaemia. Journal of Pediatrics.

[b36] Walters MC, Patience M, Leisenring W, Eckman JR, Scott JP, Mentzer WC, Davies SC, Ohene-Frempong K, Bernaudin F, Matthews DC, Storb R, Sullivan KM (1996). Bone marrow transplantation for sickle cell disease. New England Journal of Medicine.

[b37] WHO Working Group (1982). Hereditary anaemias: genetic basis, clinical features, diagnosis and treatment. Bulletin of the World Health Organization.

[b38] Williams C (1985). Neoplastic diseases of the haemopoietic system in Ibadan: preliminary report of a prospective study. The African journal of medical sciences.

[b39] Wood L, Haveman J, Juritz J, Waldman H, Hale G, Jacobs P (2009). Immunohematopoietic stem cell transplantation in Cape Town: a ten-year outcome analysis in adults. Hematology Oncology and Stem Cell Therapy.

[b40] Worlledge S, Ogiemudia SE, Thomas CO, Ikoku BN, Luzzatto L (1974). Blood group antigens and antibodies in Nigeria. Annals of Tropical Medicine and Parasitology.

[b41] Zimmerman SA, Schultz WH, Davis JS, Pickens CV, Mortier NA, Howard TA, Ware RE (2004). Sustained long-term hematologic efficacy of hydroxyurea at maximum tolerated dose in children with sickle cell disease. Blood.

